# Differential impact of COVID-19 on mental health and burnout

**DOI:** 10.1093/occmed/kqad011

**Published:** 2023-04-11

**Authors:** C Maniero, S M Ng, G Collett, T Godec, I Siddiqui, S Antoniou, A Kumar, A Janmohamed, S Nair, A Kotecha, R Khan, M Y Khanji, V Kapil, J Gupta, A K Gupta

**Affiliations:** Barts Heart Centre, St. Bartholomew’s Hospital, Barts Health NHS Trust, London EC1A 7BE, UK; William Harvey Research Institute, Queen Mary University of London, London EC1M 6BQ, UK; Barts Heart Centre, St. Bartholomew’s Hospital, Barts Health NHS Trust, London EC1A 7BE, UK; William Harvey Research Institute, Queen Mary University of London, London EC1M 6BQ, UK; William Harvey Research Institute, Queen Mary University of London, London EC1M 6BQ, UK; Wellbeing Hub, Newham Training Hub, London E15 1HP, UK; Northeast London CCG, London E15 1DA, UK; Woodgrange Medical Practice, London E7 0QH, UK; Barts Heart Centre, St. Bartholomew’s Hospital, Barts Health NHS Trust, London EC1A 7BE, UK; Wrightington, Wigan and Leigh NHS Foundation Trust, Wigan WN1 1XX, UK; St George’s University Hospitals NHS Foundation Trust, London SW17 0QT, UK; Glan Clwyd Hospital, Betsi Cadwaladr University Health Board, Wales LL18 5UJ, UK; Royal Devon and Exeter Hospital, Exeter, Devon EX2 5DW, UK; The Royal London Hospital, Barts Health NHS Trust, London E1 1BB, UK; Barts Heart Centre, St. Bartholomew’s Hospital, Barts Health NHS Trust, London EC1A 7BE, UK; William Harvey Research Institute, Queen Mary University of London, London EC1M 6BQ, UK; UCLPartners, London W1T 7HA, UK; Newham University Hospital, Barts Health NHS Trust, London E13 8SL, UK; Barts Heart Centre, St. Bartholomew’s Hospital, Barts Health NHS Trust, London EC1A 7BE, UK; William Harvey Research Institute, Queen Mary University of London, London EC1M 6BQ, UK; The Royal London Hospital, Barts Health NHS Trust, London E1 1BB, UK; South West London and St George’s Mental Health NHS Trust, London SW17 0YF, UK; Barts Heart Centre, St. Bartholomew’s Hospital, Barts Health NHS Trust, London EC1A 7BE, UK; William Harvey Research Institute, Queen Mary University of London, London EC1M 6BQ, UK; The Royal London Hospital, Barts Health NHS Trust, London E1 1BB, UK

## Abstract

**Background:**

There may be differential impact of the COVID-19 pandemic on mental health and burnout rates of healthcare professionals (HCPs) performing different roles.

**Aims:**

To examine mental health and burnout rates, and possible drivers for any disparities between professional roles.

**Methods:**

In this cohort study, online surveys were distributed to HCPs in July–September 2020 (baseline) and re-sent 4 months later (follow-up; December 2020) assessing for probable major depressive disorder (MDD), generalized anxiety disorder (GAD), insomnia, mental well-being and burnout (emotional exhaustion and depersonalization). Separate logistic regression models (at both phases) compared the risk of outcomes between roles: healthcare assistants (HCAs), nurses and midwives (nurses), allied health professionals (AHPs) and doctors (reference group). Separate linear regression models were also developed relating the change in scores to professional role.

**Results:**

At baseline (*n* = 1537), nurses had a 1.9-fold and 2.5-fold increased risk of MDD and insomnia, respectively. AHPs had a 1.7-fold and 1.4-fold increased risk of MDD and emotional exhaustion, respectively. At follow-up (*n* = 736), the disproportionate risk between doctors and others worsened: nurses and HCAs were at 3.7-fold and 3.6-fold increased risk of insomnia, respectively. Nurses also had a significantly increased risk of MDD, GAD, poor mental well-being and burnout. Nurses also had significantly worsened anxiety, mental well-being and burnout scores over time, relative to doctors.

**Conclusions:**

Nurses and AHPs had excess risk of adverse mental health and burnout during the pandemic, and this difference worsened over time (in nurses especially). Our findings support adoption of targeted strategies accounting for different HCP roles.

## Teaser

There may be differential impact of the COVID-19 pandemic on mental health and burnout rates of healthcare professionals performing different roles. This cohort study examined, at two distinct time points, the risk of depression, anxiety, insomnia, low well-being and burnout in nurses, allied health professionals (AHPs) and healthcare assistants, relative to medical doctors. We found excess risk of anxiety, depression, insomnia and burnout amongst nurses and AHPs. We also examine potential drivers that may explain these disparities.

**Key learning points**

**What is already known about this subject**

- There may be differential impact of COVID-19 on mental health and burnout rates of healthcare professionals performing different roles.
- Some studies indicate doctors are more susceptible than nurses, whereas other studies show contrasting results.
- The inconsistency can be due to single time-point assessment, narrow focus or comparing selective roles.

**What this study adds**

- We find that nurses and allied health professionals are at increased risk of adverse mental health and burnout compared to doctors, and this disparity exacerbated over time (especially for nurses).
- We also find that nurses had increased risk of COVID-19-related workplace worries which may drive the disparate mental health.

**What impact this may have on practice or policy**

- These findings may help in the prioritization and tailoring of mental health and burnout interventions for specific healthcare roles to mitigate the differential mental health impact of future pandemics.

## INTRODUCTION

The increased demand on the healthcare workforce because of the coronavirus disease 2019 (COVID-19) pandemic has had a profound effect on the mental health of healthcare professionals (HCPs). Due to the potential for reduced quality of patient care and work absence [1–3], we must identify the risk factors for adverse mental health among HCPs and the mechanisms to support them under pandemic conditions [4].

Factors such as age, working in junior positions, being parents of dependent children and having an infected family member are associated with poorer mental health in HCPs during pandemics [5]. It is debatable whether all HCPs are similarly affected: a few studies have shown that physicians (doctors) were more affected than those in nursing profession [6–8], whereas other studies have shown contrasting results [9–15]. The inconsistency in study findings can be due to small studies, single time-point assessment, narrow focus or comparing selective roles [6–11,15]. Additionally, existing studies generally lack reporting the potential drivers of this differential impact, if any. Moreover, relatively underexamined roles such as allied health professionals (AHPs) and healthcare assistants (HCAs) must be investigated to inform role-tailored mental health interventions.

Addressing these shortcomings, the primary objective of this cohort study (part of the COVID-19 Disease and Physical and Emotional Wellbeing of Healthcare Professionals project; CoPE-HCP) [16] was to examine the relationship between different healthcare roles and various mental health outcomes at two distinct periods during the COVID-19 pandemic. Supplementing this, we examined the relationship between different healthcare roles and the change in mental health and burnout scores over the 4-month study period. Our secondary objective was to examine the relationship between healthcare roles and specific COVID-19-related worries, as a possible driver. We hypothesized a differential impact on mental health across healthcare roles and these differences would increase over time.

## METHODS

The project protocol has been published containing details of the project methodology [16] (also available on clinicaltrials.gov; NCT04433260). The study was approved by the Cambridge East Research Ethics Committee (20/EE/0166). Informed (digital) consent was obtained from all participants.

Participant recruitment was facilitated via open invitation of internally controlled e-mail lists of NHS participating institutions, and Queen Mary University of London for control sample (wider project).

This study involved a series of online surveys. The baseline survey was conducted between July and September 2020 which corresponded to the trough of the first wave of COVID-19 and the easing of the first UK lockdown.

The baseline survey gathered demographic information, current mental health and physical health diagnosis (multiple-choice closed-ended item or stating ‘other’) and domains including professional role, work environment and COVID-19-related worries: worry about their own health, risk and inadequate PPE, risk of family catching COVID-19, inadequate training to deal with COVID-19-related tasks, inadequate supervision and redeployment. The worry items were rated from 1 to 5: ‘never’, ‘rarely’, ‘sometimes’, ‘often’ and ‘always’.

The baseline survey also included validated mental health assessments: Patient Health Questionnaire-9 (PHQ-9) [17], Generalized Anxiety Disorder-7 (GAD-7) [18] and Insomnia Sleep Index (ISI) [19] assessing probable major depressive disorder (MDD), generalized anxiety disorder (GAD) and clinical insomnia, respectively. Mental well-being was assessed using the Short Warwick-Edinburgh Mental Wellbeing Scale (SWEMWBS) [20]. It also included two abbreviated items from the Maslach Burnout Inventory [21] assessing emotional exhaustion and depersonalization, respectively (Table 1).

**Table 1.** Outcome variables and respective assessment tools

Variables	Assessment tool	Cut-off score
Probable major depressive disorder	Patient Health Questionnaire-9 (PHQ-9)	A score of ≥10 indicates the presence of probable major depressive disorder.
Probable generalized anxiety disorder	Generalized Anxiety Disorder-7 (GAD-7)	A score of ≥10 indicates the presence of probable generalized anxiety disorder.
Probable clinical insomnia	Insomnia Severity Index (ISI)	A score of ≥15 indicates the presence of probable clinical insomnia.
Possible or probable low mental well-being	Short Warwick-Edinburgh Mental Wellbeing Scale (SWEMWBS)	A score of ≤20 indicates possible or probable low mental well-being.
Burnout (emotional exhaustion)	Emotional Exhaustion Item from the abbreviated 2-question summative score (EEDP2Q).	A score of ≥4 (‘a few times a month’) indicates emotional exhaustion.
Burnout (depersonalization)	Depersonalization Item from the abbreviated 2-question summative score (EEDP2Q).	A score of ≥4 (‘a few times a month’) indicates depersonalization.

At the end of the baseline survey, participants were asked if they consent to receiving follow-up surveys. The follow-up survey was conducted 4 months later (December 2020) and corresponded with the second UK national lockdown due to the rise in COVID-19 cases during the winter of 2020. The follow-up survey contained the same mental health assessments.

Statistical analyses were conducted using STATA v17.0. Of 2100 available records, only self-identified HCPs were included and categorized into four groups: doctors (regardless of training level: senior doctor, higher specialist trainee, clinical fellow, core trainee or foundation doctor), HCAs (including phlebotomists, porters, cleaners), nurses (including midwives) and AHPs (pharmacists, occupational therapists, physiotherapists, radiographers). Descriptive statistics for sociodemographic characteristics were presented for each role as the number within each group and percentage. For follow-up, only participants who had provided a baseline survey were included.

Outcome-based analysis was performed on a complete case basis, excluding subjects from a particular analysis if they had not responded to items relevant to the primary outcomes. Missingness for primary outcomes was minimal (<10%).

The following validated cut-offs were used for primary outcomes. Since cut-offs do not provide official clinical diagnosis, we define our outcomes as ‘probable’. For the PHQ-9, probable MDD is defined as ≥10 [17]. For the GAD-7, probable GAD is defined as ≥10 [18]. For the ISI, clinical insomnia is defined as ≥15 [19]. Possible depression/anxiety (or low well-being) on the SWEMWBS is indicated by scoring ≤20 [20]. Regarding burnout, scoring ≥4 indicated emotional exhaustion and depersonalization for both respective scales [21] (Table 1).

We performed separate logistic regression analyses for each outcome (at baseline and follow-up) between the four HCP roles. Crude and adjusted odds ratios (ORs; with 95% confidence intervals [CIs] and *P-*values for the global trend across roles) were calculated with doctors as the reference group. Models were adjusted for *a priori* confounders: age, gender, time since COVID-19 peak, education, relationship status, number of people living in the household, current diagnosis of mental health condition (yes/no), current diagnosis of physical health condition (yes/no) and full-time/part-time work status. The models were not adjusted for COVID-19-related worries due to risk of over-adjustment.

Due to potential bias at follow-up, we conducted chi-square analyses on demographic characteristics and mental health, in baseline-only participants compared to cohort participants.

Separate linear regression analyses were performed to relate professional role to the change in PHQ-9, GAD-7, ISI, SWEMWBS and combined burnout domain scores from baseline to follow-up. The change in scores was calculated by subtracting the baseline score from the follow-up score for each participant. Crude and adjusted coefficients (with 95% CIs and *P-*values for global trend) were calculated with doctors as the reference group. Models were adjusted as above.

Separate logistic regression analyses were then performed to relate COVID-related worries (assessed at baseline) to each HCP role. Each response was dichotomized into ‘worried’ (‘always’, ‘often’ and ‘sometimes’) or ‘not worried’ (‘rarely’ and ‘never’). Crude and adjusted ORs (with 95% CIs and *P-*values) were calculated with doctors as the reference group. Models were adjusted as above.

As a crude indicator of COVID-19-related worries as a potential driver, a Pearson’s correlation analysis was conducted relating COVID-19-related worries to raw mental health scores at baseline and follow-up. Listwise deletion was used for the correlation analysis.

## RESULTS

The baseline surveys were received between 24 July 2020 and 2 October 2020 (51% were received by 1 September 2020). The follow-up surveys were received between 22 January 2021 and 13 March 2021 (55% were received by 28 January 2021).

Of the 1537 participants at baseline, most were UK-based (84%) and, of these, most were based in London (70%). Regarding roles, 42% (651) were doctors, 15% (223) were HCAs, 24% (368) were nurses and 19% (295) were AHPs. Most were aged between 36 and 50 years (41%) and were predominantly females (70%). Generally, doctors (52%) and AHPs (48%) had higher education attainment (Master’s degree or PhD), compared to HCAs (21%) and nurses (21%). The proportions working full-time or part-time did not vary considerably across roles (Supplementary Table 1, available as Supplementary data at *Occupational Medicine* Online).

Most participants worked 35–45 h per week (48%), but a larger proportion of doctors worked >45 h per week (38%), compared to HCAs (9%), nurses (17%) and AHPs (15%). Relatively fewer HCAs (38%) reported appropriate PPE use training compared to doctors (59%), nurses (61%) and AHPs (49%). Workplace practices were reported as a source of stress by 76% of doctors, 69% of HCAs, 82% of nurses and 78% of AHPs (Supplementary Table 2, available as Supplementary data at *Occupational Medicine* Online).

At baseline (Supplementary Table 3a, available as Supplementary data at *Occupational Medicine* Online), a higher proportion of nurses reported symptoms suggestive of COVID-19 in the preceding few months (35%), compared with doctors (32%), HCAs (26%) and AHPs (29%). A higher proportion of nurses also reported a positive COVID-19 test (22%), compared with doctors (17%), HCAs (16%) and AHPs (19%).

At follow-up (Supplementary Table 3b, available as Supplementary data at *Occupational Medicine* Online), 40% of nurses reported symptoms suggestive of COVID-19 since the start of the pandemic, compared with doctors (38%), HCAs (32%) and AHPs (32%). Consistently, evidence of positive COVID-19 tests also increased at follow-up in all roles (*n* = 736) with nurses (28%) being higher compared to doctors (24%), HCAs (19%) and AHPs (24%).

At baseline (*n* = 1537; Table 2), the rates of probable mental health issues were considerable: 25% had probable MDD, 20% had GAD and 16% had clinical insomnia. Regarding well-being, 25% had possible depression/anxiety. Regarding burnout, emotional exhaustion and depersonalization were apparent in 42% and 13% of respondents, respectively.

**Table 2.** Baseline outcomes by HCP role.

Outcome	HCP role	Events (%)	Crude ORs (95% CI)	*P*	Adjusted ORs (95% CI) ^*^	*P* ^*^
Major depressive disorder	Medical doctor	102 (17)	Reference	<0.001	Reference	0.003
	HCA or other	66 (32)	2.34 (1.63–3.36)		1.53 (0.96–2.42)	
	Nurse and midwives	105 (30)	2.11 (1.55–2.89)		1.90 (1.32–2.73)	
	AHPs	81 (29)	1.98 (1.42–2.77)		1.72 (1.19–2.48)	
Generalised anxiety disorder	Medical doctor	92 (15)	Reference	0.003	Reference	0.400
	HCA or other	53 (26)	1.93 (1.31–2.83)		1.12 (0.68–1.85)	
	Nurse and midwives	78 (23)	1.59 (1.14–2.23)		1.37 (0.92–2.02)	
	AHPs	61 (22)	1.52 (1.06–2.18)		1.28 (0.86–1.91)	
Clinical insomnia	Medical doctor	66 (11)	Reference	<0.001	Reference	<0.001
	HCA or other	40 (20)	1.99 (1.29–3.06)		1.69 (1.00–2.87)	
	Nurse and midwives	89 (26)	2.79 (1.96–3.96)		2.54 (1.70–3.79)	
	AHPs	33 (12)	1.08 (0.69–1.69)		0.95 (0.59–1.52)	
Possible depression or anxiety (SWEMWBS)	Medical doctor	117 (20)	Reference	0.001	Reference	0.341
	HCA or other	65 (33)	1.95 (1.36–2.79)		1.30 (0.83–2.04)	
	Nurse and midwives	94 (28)	1.54 (1.12–2.10)		1.37 (0.96–1.95)	
	AHPs	73 (27)	1.46 (1.04–2.04)		1.20 (0.84–1.71)	
Burnout (Emotional Exhaustion)	Medical doctor	238 (41)	Reference	0.104	Reference	0.113
	HCA or other	76 (38)	0.90 (0.65–1.26)		0.94 (0.63–1.41)	
	Nurse and midwives	135 (40)	0.98 (0.75–1.29)		1.08 (0.79–1.47)	
	AHPs	131 (48)	1.36 (1.02–1.81)		1.42 (1.04–1.95)	
Burnout (Depersonalisation)	Medical doctor	91 (16)	Reference	0.067	Reference	0.041
	HCA or other	16 (8)	0.48 (0.27–0.83)		0.36 (0.18–0.74)	
	Nurse and midwives	44 (13)	0.82 (0.56–1.21)		0.90 (0.57–1.40)	
	AHPs	35 (13)	0.80 (0.53–1.22)		0.77 (0.49–1.21)	

* Adjusted for age, gender, time since COVID peak, highest level of education, relationship status, number living in household, current diagnosis of mental health condition, current diagnosis of physical health condition, and full-time/part-time working status.

*P* values are for global trend across all roles relative to medical doctors.

There are differing levels of missing data for each outcome, and for each model. Out of 1537 subjects for whom we have been able to define a HCW role:

• Major depressive disorder – 1434 were included in all analyses. 601 were medical doctors, 204 were HCAs or other, 348 were nurses and midwives, 281 were AHPs.

• Generalised anxiety disorder - 1429 were included in all analyses. 597 were medical doctors, 204 were HCAs or other, 347 were nurses and midwives, 281 were AHPs.

• Clinical insomnia - 1418 were included in all analyses. 595 were medical doctors, 201 were HCAs or other, 245 were nurses and midwives, 277 were AHPs.

• Possible or probable depression or anxiety (SWEMWBS) – 1393 were included in all analyses. 584 were medical doctors, 198 were HCAs or other, 338 were nurses and midwives, 273 were AHPs.

• Burnout (Emotional Exhaustion) - 1386 were included in all analyses. 583 were medical doctors, 198 were HCAs or other, 334 were nurses and midwives, 271 were AHPs.

• Burnout (Depersonalisation) – As above for emotional exhaustion.

At baseline (Table 2), compared to doctors, nurses and AHPs had 90% (adjusted OR 1.90, 95% CI 1.32–2.73) and 72% (1.72, 1.19–2.48) greater risk of MDD, respectively. Nurses also had 2.5-fold (2.54, 1.70–3.79) excess risk of clinical insomnia. There were significant differences in burnout rates too: compared to doctors, AHPs had a 42% greater risk of emotional exhaustion (1.42, 1.04–1.95), whereas HCAs had a 64% lower risk for depersonalization (0.36, 0.18–0.74).

At follow-up (Table 3; *n* = 736; 48% of baseline sample), the difference in risk of various probable mental health issues between doctors and other HCPs appeared to worsen: compared to doctors, nurses (1.87, 1.14–3.07) and AHPs (1.89, 1.16–3.07) had about a 1.9-fold significantly greater risk of MDD. HCAs and nurses were about 3.6- (3.57, 1.66–7.66) and 3.7-fold (3.68, 2.00–6.80) more likely to have clinical insomnia, compared to doctors. Nurses were also two times more likely to have GAD (2.00, 1.15–3.45), and about 1.7-fold more likely to have possible depression/anxiety according to well-being measure (1.68, 1.05–2.70), emotional exhaustion (1.66, 1.07–2.58) and 2-fold more likely to have depersonalization (1.95, 1.16–3.27).

**Table 3.** Follow-up (4 months later) outcomes by HCP role.

Outcome	HCP role	Events (%)	Crude ORs (95% CI)	*P*	Adjusted ORs (95% CI)^*^	*P* ^*^
Major depressive disorder	Medical doctor	50 (20)	Reference	0.005	Reference	0.036
	HCA or other	35 (33)	2.04 (1.22–3.40)		1.70 (0.89–3.25)	
	Nurse and midwives	61 (32)	1.91 (1.24–2.95)		1.87 (1.14–3.07)	
	AHPs	53 (32)	1.91 (1.22–3.00)		1.89 (1.16–3.07)	
Generalised anxiety disorder	Medical doctor	37 (15)	Reference	0.007	Reference	0.088
	HCA or other	30 (29)	2.38 (1.37–4.12)		1.78 (0.87–3.62)	
	Nurse and midwives	48 (25)	1.97 (1.22–3.17)		2.00 (1.15–3.45)	
	AHPs	33 (20)	1.46 (0.87–2.44)		1.36 (0.78–2.39)	
Clinical insomnia	Medical doctor	24 (10)	Reference	<0.001	Reference	<0.001
	HCA or other	26 (25)	3.18 (1.73–5.86)		3.57 (1.66–7.66)	
	Nurse and midwives	47 (25)	3.11 (1.83–5.31)		3.68 (2.00–6.80)	
	AHPs	20 (12)	1.31 (0.70–2.45)		1.30 (0.67–2.55)	
Possible or probable depression or anxiety (SWEMWBS)	Medical doctor	58 (23)	Reference	0.002	Reference	0.112
	HCA or other	44 (42)	2.45 (1.51–3.99)		1.84 (1.00–3.40)	
	Nurse and midwives	65 (34)	1.74 (1.14–2.65)		1.68 (1.05–2.70)	
	AHPs	49 (30)	1.40 (0.90–2.18)		1.32 (0.82–2.13)	
Burnout (Emotional Exhaustion)	Medical doctor	97 (39)	Reference	0.295	Reference	0.157
	HCA or other	46 (45)	1.31 (0.82–2.09)		1.33 (0.74–2.39)	
	Nurse and midwives	89 (47)	1.42 (0.97–2.08)		1.66 (1.07–2.58)	
	AHPs	74 (45)	1.29 (0.86–1.91)		1.23 (0.80–1.89)	
Burnout (Deperson-alisation)	Medical doctor	52 (21)	Reference	0.213	Reference	0.011
	HCA or other	19 (19)	0.88 (0.49–1.58)		0.88 (0.41–1.87)	
	Nurse and midwives	50 (27)	1.38 (0.89–2.16)		1.95 (1.16–3.27)	
	AHPs	30 (18)	0.85 (0.51–1.40)		0.81 (0.47–1.41)	

* Adjusted for age, gender, time since COVID peak, highest level of education, relationship status, number living in household, current diagnosis of mental health condition, current diagnosis of physical health condition, and part-time/full-time working status.

*P* values are for global trend across all roles relative to medical doctors.

There are differing levels of missing data for each outcome, and for each model. Out of 736 subjects for whom we have been able to define a HCP role, and who had both a baseline and follow-up survey response:

• Major depressive disorder – 716 were included in all analyses. 254 were medical doctors, 105 were HCAs or other, 191 were nurses and midwives, 166 were AHPs.

• Generalised anxiety disorder - 715 were included in all analyses. 254 were medical doctors, 104 were HCAs or other, 191 were nurses and midwives, 166 were AHPs.

• Clinical insomnia - 714 were included in all analyses. 253 were medical doctors, 104 were HCAs or other, 191 were nurses and midwives, 166 were AHPs.

• Possible or probable depression or anxiety (SWEMWBS) – 712 were included in all analyses. 252 were medical doctors, 104 were HCAs or other, 190 were nurses and midwives, 166 were AHPs.

• Burnout (Emotional Exhaustion) - 709 were included in all analyses. 252 were medical doctors, 102 were HCAs or other, 189 were nurses and midwives, 166 were AHPs.

• Burnout (Depersonalisation) – As above for emotional exhaustion.

Chi-square analysis indicated no significant differences in mental health of follow-up participants (*n* = 736) compared to baseline-only participants (*n* = 801). Demographic characteristics were balanced, except for ethnicity (*P* < 0.01), gender identity (*P* < 0.01) and number living in the household (*P* < 0.01): the follow-up sample had larger proportions of female and White participants, and fewer people living in the household compared to baseline-only participants.

Table 4 reports the change in mental health and burnout scores over the study period in the cohort (Table 4; *n* = 736). Compared to doctors, nurses had significantly increased change in GAD-7 (1.36 [0.46 to 2.26]; *P* < 0.01) and combined emotional exhaustion and depersonalization scores (1.15 [0.59 to 1.71]; P < 0.001) over the study period. Nurses and midwives also had significantly reduced change in mental well-being scores (−1.17 [−1.94 to −0.40]; *P* < 0.01) over the study period. No significant associations were observed for other roles. Supplementary Table 4 (available as Supplementary data at *Occupational Medicine* Online) displays mean values for change in PHQ-9, GAD-7, ISI, SWEMWBS and combined emotional exhaustion and depersonalization scores, stratified by roles.

**Table 4.** Separate linear regressions for the association between change in mental health, wellbeing, and burnout scores from baseline to follow-up and professional roles (medical doctors as reference group).

Crude					Adjusted		
		Coefficient	95% Confidence Intervals	*P* Value	Coefficient ^*^	95% Confidence Intervals ^*^	*P* value ^*^
**PHQ-9** **(n = 695)**	Medical doctor	-	-	-	-	-	-
	HCA or other	-0.89	-1.93 to 0.15	0.09	0.09	-1.15 to 1.32	0.89
	Nurse and midwives	0.03	-0.82 to 0.89	0.94	0.27	-0.66 to 1.20	0.57
	AHPs	-0.54	-1.42 to 0.35	0.23	-0.43	-1.35 to 0.48	0.36
**GAD-7** **(n = 694)**	Medical doctor	-	-	-	-	-	-
	HCA or other	0.12	-0.88 to 1.12	0.82	0.66	-0.53 to 1.86	0.28
	Nurse and midwives	1.08	0.25 to 1.90	0.01	1.36	0.46 to 2.26	< 0.01
	AHPs	0.55	-0.30 to 1.40	0.21	0.67	-0.22 to 1.56	0.14
**ISI** **(n = 692)**	Medical doctor	-	-	-	-	-	-
	HCA or other	-0.37	-1.49 to 0.75	0.52	-0.04	-1.40 to 1.33	0.96
	Nurse and midwives	-0.07	-0.98 to 0.85	0.89	0.31	-0.71 to 1.33	0.55
	AHPs	-0.50	-1.45 to 0.45	0.31	-0.29	-1.30 to 0.72	0.57
**SWEMWBS** **(n = 687)**	Medical doctor	-	-	-	-	-	-
	HCA or other	-0.51	-1.36 to 0.34	0.24	-0.55	-1.57 to 0.47	0.29
	Nurse and midwives	-1.15	-1.85 to -0.45	0.001	-1.17	-1.94 to -0.40	< 0.01
	AHPs	-0.58	-1.31 to 0.14	0.12	-0.48	-1.24 to 0.28	0.22
**Combined burnout domains** **(n = 684)**	Medical doctor	-	-	-	-	-	-
	HCA or other	0.01	-0.60 to 0.63	0.96	-0.07	-0.82 to 0.67	0.85
	Nurse and midwives	0.95	0.45 to 1.46	< 0.001	1.15	0.59 to 1.71	< 0.001
	AHPs	0.32	-0.20 to 0.84	0.23	0.34	-0.22 to 0.89	0.24

*Note.* Crude and adjusted coefficients provided.

^*^Adjusted for age, gender, time since COVID peak, highest level of education, relationship status, number living in household, current diagnosis of mental health condition, current diagnosis of physical health condition, and part-time/full-time working status.

At baseline (*n* = 1403; Table 5), compared to doctors, nurses were about 1.5-fold more likely to worry about their own health and well-being (1.46, 1.03–2.07), and lack of training provisions for dealing with COVID-19-related tasks (1.43, 1.06–1.94). Nurses were also 2-fold more likely to worry about provision of adequate supervision in workplace (1.96, 1.44–2.67) or being redeployed (2.04, 1.50–2.76). In contrast, HCAs were 47% less likely than doctors to worry about their family catching COVID-19 due to their work (0.53, 0.33–0.84) and 37% less likely to worry about inadequate training to deal with COVID-19-related jobs (0.63, 0.42–0.93), whereas AHPs were 28% less likely to worry about provision of PPE (0.72, 0.53–0.97). Both doctors and nurses shared similar level of concern about PPE and family catching COVID-19.

**Table 5.** COVID-related worries by HCP role with medical doctors as the reference group (n = 1403).

	HCP role	Events (%)	Crude ORs (95% CI)	*P*	Adjusted ORs (95% CI)^*^	*P* ^*^
**Worry about your health as a result of Covid-19 pandemic**	Medical doctor	400 (68)	Reference	0.002	Reference	0.174
	HCA or other	158 (79)	1.76 (1.20–2.58)		1.31 (0.84–2.05)	
	Nurse and midwives	263 (77)	1.58 (1.16–2.14)		1.46 (1.03–2.07)	
	AHPs	191 (70)	1.06 (0.78–1.45)		1.20 (0.86–1.68)	
**Worry about being at greater risk due to not having adequate PPE**	Medical doctor	304 (52)	Reference	0.057	Reference	0.064
	HCA or other	92 (46)	0.79 (0.57–1.09)		0.69 (0.46–1.02)	
	Nurse and midwives	173 (51)	0.96 (0.73–1.25)		0.97 (0.72–1.32)	
	AHPs	117 (43)	0.69 (0.52–0.92)		0.72 (0.53–0.97)	
**Worry about your family catching COVID-19 due to your work**	Medical doctor	484 (83)	Reference	0.098	Reference	0.054
	HCA or other	153 (77)	0.69 (0.47–1.02)		0.53 (0.33–0.84)	
	Nurse and midwives	272 (80)	0.84 (0.60–1.18)		0.83 (0.56–1.22)	
	AHPs	209 (76)	0.67 (0.48–0.96)		0.76 (0.52–1.11)	
**Worry about not having adequate training to deal with COVID-19 related jobs**	Medical doctor	298 (51)	Reference	0.004	Reference	<0.001
	HCA or other	87 (44)	0.75 (0.54–1.03)		0.63 (0.42–0.93)	
	Nurse and midwives	202 (59)	1.41 (1.08–1.85)		1.43 (1.06–1.94)	
	AHPs	137 (50)	0.96 (0.72–1.28)		0.95 (0.70–1.29)	
**Worry about not having adequate supervision in workplace**	Medical doctor	197 (34)	Reference	0.001	Reference	<0.001
	HCA or other	82 (41)	1.38 (0.99–1.91)		1.14 (0.76–1.70)	
	Nurse and midwives	161 (47)	1.77 (1.35–2.33)		1.96 (1.44–2.67)	
	AHPs	103 (38)	1.19 (0.88–1.60)		1.14 (0.83–1.57)	
**Worry about being redeployed**	Medical doctor	233 (40)	Reference	<0.001	Reference	<0.001
	HCA or other	85 (43)	1.12 (0.81–1.55)		1.02 (0.69–1.51)	
	Nurse and midwives	190 (56)	1.91 (1.46–2.50)		2.04 (1.50–2.76)	
	AHPs	118 (43)	1.14 (0.85–1.53)		1.05 (0.77–1.43)	

^*^Adjusted for age, gender, time since COVID peak, highest level of education, relationship status, number living in household, current diagnosis of mental health condition, current diagnosis of physical health condition, and part-time/full-time working status.

Note. Analyses are based on worry outcomes presented as binary events when subjects answer either “often” or “always”.

***P* values** are for global trend across all roles relative to medical doctors.

Note on missing data: Out of 1537 subjects for whom we have been able to define a HCW role, 1403 were included in these analyses, dropping 134 subjects with no outcome data on worries relating to COVID-19. Participants categorised as: Medical doctors = 587, HCAs or other = 200, Nurses and midwives = 341, AHPs = 275.

Significant associations were observed between COVID-19-related worry items and raw mental health scores at baseline (all coefficients *P* < 0.01) (*n* = 1386; Supplementary Table 5, available as Supplementary data at *Occupational Medicine* Online), and between each baseline worry item and follow-up raw mental health scores (*n* = 685; all coefficients *P* < 0.01 except for between depersonalization and worry about redeployment, *P* < 0.05).

## DISCUSSION

Despite the COVID-19 pandemic having a huge impact on the mental health of both HCPs and non-HCPs (albeit disproportionately higher burnout amongst HCPs) [22], our study demonstrates mental health and burnout disparities across different HCP roles. Compared to doctors, nurses were 1.9-fold and 2.5-fold more likely to have probable MDD and clinical insomnia, respectively, at baseline. Similarly, AHPs were 1.7 times and 1.4 times more likely to have probable MDD and emotional exhaustion (burnout), respectively. In contrast, compared to doctors, HCAs were 64% less likely to have depersonalization (burnout). These findings were consistent 4 months later. The follow-up analysis also showed an increase in the risk difference, and strength of association: compared to doctors, nurses were 87% more likely to have probable MDD, 3.7 times more likely to have clinical insomnia, 2-fold more likely to have GAD, 68% more likely to have low mental well-being and 66% and 95% more likely to have emotional exhaustion and depersonalization, respectively. The significant increased risk of probable GAD, burnout and low mental well-being in nurses observed at follow-up (but not baseline) was supported by our linear regression models: nurses had significantly worsened GAD-7, SWEMWBS and burnout symptoms over time, relative to doctors. These findings highlight that the mental health impact of the COVID-19 pandemic disproportionately affects nurses on multiple domains, relative to doctors.

Furthermore, nurses had greater risk of worry regarding several work-related and COVID-19-related aspects, relative to doctors, which may explain the disparities in mental health and indicates that support strategies must be modified for specific roles to mitigate the mental health and burnout impact on HCPs. For example, providing adequate training and supervision, and adequate staffing in all areas to mitigate redeployment worries, may help protect against adverse mental health in nurses and midwives during pandemics.

These findings could be explained by differential susceptibility (e.g. varying levels of resilience [23–26]) to adverse mental health between roles. However, a Japanese study found, while nurses were more likely than doctors to have MDD, there were no significant differences between occupation on resilience measures, despite resilience scores predicting MDD rates across the total sample [27]. We speculate that specific workplace factors relevant to the role are a more likely explanation (i.e. differential exposures to health hazards: the time spent in patient-facing roles and exposure to COVID-19) [28]. Nurses often spend more time in direct patient-facing roles, with tasks involving greater proximity to patients and emergency care, relative to doctors. Indeed, we observed more nurses testing positive for COVID-19 and missing more days due to work, relative to doctors, which could indicate increased exposure to COVID-19 in patient-facing settings.

While AHPs might not spend as much time in a patient-facing setting relative to HCAs and nurses, we observed that AHPs were at significantly higher risk of MDD than doctors at both phases. Similar observations have been made previously [9]. AHPs may have had increased time facing patients compared to before the pandemic, but may experience additive stressors such as medication shortages (in the case of pharmacists) and triage which are not normally encountered [29]. Therefore, tailored support measures should be implemented for AHPs where their responsibilities drastically change compared to before the pandemic.

Regarding strengths, this is amongst the first cohort studies to evaluate the risk of adverse mental health between HCP roles during the pandemic. To our knowledge, just one study compared the mental health impact between HCP roles at two separate points during the pandemic, but the assessment of mental health was relatively narrow [14]. Drawing on this, a second strength is our inclusion of relatively underexamined issues such as burnout and insomnia. Finally, a unique aspect is that we evaluated for the potential underlying cause of the differential mental health and found important workplace- and worry-related differences between roles.

However, there are limitations. First, while the validated mental health assessments are appropriate for large samples, these are less accurate than face-to-face psychiatric assessment. Secondly, there are comparisons within groups which were not analysed: within AHPs, we do not distinguish between occupational therapists or pharmacists which might reduce the specificity of any interventions/policy changes made (similar assertions can be made when comparing nurses to midwives). One could argue that senior doctors (e.g. consultants) and other doctors should not be combined due to differences in functions and powers, but no significant differences between these two groups were observed regarding mental health (Supplementary Tables 6a and 6b, available as Supplementary data at *Occupational Medicine* Online). A useful avenue of inquiry would also be comparing between departments (e.g. oncology versus intensive care units). Third, we did not assess domains such as moral injury [30] which may have provided further insight into the disproportionate mental health. Fourth, we cannot rule out that our participants, especially those who remained at follow-up, were more likely to exhibit mental health issues than non-participants, although we observed no major differences in profile between those who dropped out and those who remained at follow-up. That said, follow-up participants may be less likely to exhibit mental health issues due to the ‘healthy worker’ effect. Finally, the pandemic conditions changed rapidly during 2021 and 2022, and there are additional stressors such as the cost-of-living and health service budgeting crises, as well as new exposures (e.g. Mpox/Monkeypox), which might disproportionately impact different roles. An assessment of job attrition and a re-assessment of mental health between roles would be valuable in this context.

Overall, this study demonstrates that, on multiple domains, the mental health and burnout of nurses during the COVID-19 pandemic are more adversely impacted than in doctors. By follow-up, nurses were more likely to have probable MDD, GAD, clinical insomnia, low mental well-being and burnout. AHPs may also be at increased risk of probable MDD compared to doctors, which was sustained across the study period. These findings may help in the prioritization and tailoring of well-being interventions for specific healthcare roles to mitigate the differential mental health impact of the COVID-19 pandemic.

## Supplementary Material

kqad011_suppl_Supplementary_Tables

## Data Availability

Anonymized data, data dictionary and survey materials will be made available upon request (contact corresponding and senior author: A.K.G.). Study protocol is available at https://doi.org/10.3389/fpsyg.2021.616280.
