# Supplementary material for: Differential impact of COVID-19 on mental health and burnout
Source: Occup Med (Lond). 2023 Apr 11;74(1):45–52. doi: 10.1093/occmed/kqad011 (PMC10875923; doi:10.1093/occmed/kqad011)
Supplement: kqad011_suppl_Supplementary_Tables [file kqad011_suppl_supplementary_tables.docx]

| **Supplemental Table 1.** Baseline demographic, social and educational characteristics of all participants. | | | | | | |
| --- | --- | --- | --- | --- | --- | --- |
| **Characteristic** | **Response** | **Medical Doctor**  **N = 651 (%)** | **HCA or other**  **N = 223 (%)** | **Nurses or Midwives**  **N = 368 (%)** | **AHP or Pharmacist**  **N = 295 (%)** | **Overall**  **N = 1537 (%)** |
| **Age** | 18-25 years | 22 (3.4) | 16 (7.2) | 17 (4.6) | 18 (6.1) | 73 (4.7) |
|  | 26-35 years | 168 (25.8) | 50 (22.4) | 68 (18.5) | 95 (32.2) | 381 (24.8) |
|  | 36-50 years | 315 (48.4) | 74 (33.2) | 136 (37.0) | 102 (34.6) | 627 (40.1) |
|  | 51-60 years | 118 (18.1) | 65 (29.1) | 120 (32.6) | 58 (19.7) | 361 (23.5) |
|  | 61+ years | 28 (18.1) | 18 (8.1) | 27 (7.3) | 22 (7.5) | 95 (6.2) |
| **Ethnicity** | White | 325 (49.9) | 158 (70.1) | 276 (75.0) | 246 (83.4) | 1005 (65.4) |
|  | Asian | 256 (39.3) | 27 (12.1) | 38 (10.3) | 29 (9.8) | 350 (22.8) |
|  | Black | 11 (1.7) | 22 (10.0) | 30 (8.2) | 9 (3.1) | 72 (4.7) |
|  | Mixed | 24 (3.7) | 4 (1.8) | 15 (4.1) | 4 (1.4) | 47 (3.1) |
|  | Other | 23 (3.5) | 8 (3.6) | 5 (1.4) | 2 (0.7) | 38 (2.5) |
|  | Prefer not to say | 12 (1.8) | 4 (1.8) | 4 (1.1) | 5 (1.7) | 25 (1.6) |
| **Gender identity** | Female | 368 (56.5) | 181 (81.2) | 309 (84.0) | 223 (75.6) | 1081 (70.3) |
|  | Male | 274 (42.1) | 36 (16.1) | 56 (15.2) | 69 (23.4) | 435 (28.3) |
|  | Prefer not to say | 6 (0.9) | 2 (0.9) | 2 (0.5) | 3 (1.0) | 13 (0.8) |
|  | Prefer to self-define | 3 (0.46) | 4 (1.8) | 1 (0.3) | 0 (0.0) | 8 (0.5) |
| **Relationship status** | Divorced | 18 (2.8) | 11 (4.9) | 18 (4.9) | 5 (1.7) | 52 (3.4) |
|  | I prefer not to disclose | 15 (2.3) | 9 (4.0) | 12 (3.3) | 5 (1.7) | 41 (2.7) |
|  | Married/Living with partner or family | 467 (71.7) | 135 (60.5) | 237 (64.4) | 186 (63.1) | 1025 (66.7) |
|  | Other | 14 (2.2) | 13 (5.8) | 14 (3.8) | 8 (2.7) | 49 (3.2) |
|  | Single | 137 (21.0) | 55 (24.7) | 87 (23.6) | 91 (30.8) | 370 (24.1) |
| **Number living in household** | 1 | 80 (12.3) | 25 (11.2) | 55 (14.9) | 44 (14.9) | 204 (13.3) |
|  | 2 | 172 (26.4) | 80 (35.9) | 114 (31.0) | 108 (36.6) | 474 (30.8) |
|  | 3-5 | 353 (54.2) | 108 (48.4) | 186 (50.5) | 136 (46.1) | 783 (50.9) |
|  | 6 or more | 46 (7.1) | 10 (4.5) | 13 (3.5) | 7 (2.4) | 76 (4.9) |
| **Highest qualification** | A-levels | 1 (0.2) | 82 (36.8) | 13 (3.5) | 11 (3.7) | 107 (7.0) |
|  | Bachelors degree/diploma | 254 (39.0) | 73 (32.7) | 258 (70.1) | 137 (46.4) | 722 (47.0) |
|  | Master's degree or PhD | 336 (51.6) | 47 (21.1) | 76 (20.7) | 141 (47.8) | 600 (39.0) |
|  | Other | 60 (9.2) | 21 (9.4) | 21 (5.7) | 6 (2.0) | 108 (7.0) |
| **Full-time/part-time** | Full-time (>35 hours) | 517 (79.4) | 169 (75.8) | 286 (77.7) | 226 (76.6) | 1198 (77.9) |
|  | Part-time | 134 (20.6) | 54 (24.2) | 82 (22.3) | 69 (23.4) | 339 (22.1) |
|  | | | | | | |

| Supplemental Table 2. Clinical experience, perceptions of re-training, redeployment rates and use of PPE in all participants. | | | | | | |
| --- | --- | --- | --- | --- | --- | --- |
| Question | **Response** | **Medical doctor**  **N = 651 (%)** | **HCA or other**  **N = 223 (%)** | **Nurses or midwives**  **N = 368 (%)** | **AHP or Pharmacist**  **N = 295 (%)** | **Overall**  **N = 1537 (%)** |
| Years of full-time clinical experience | <3 | 63 (9.7) | 28 (12.6) | 23 (6.3) | 44 (14.9) | 158 (10.3) |
|  | 3-8 | 130 (20.0) | 33 (14.8) | 69 (18.8) | 73 (24.7) | 305 (19.8) |
|  | 8-14 | 133 (20.4) | 31 (13.9) | 55 (14.9) | 52 (17.6) | 271 (17.6) |
|  | 15-20 | 110 (16.9) | 21 (9.4) | 62 (16.8) | 42 (14.2) | 235 (15.3) |
|  | >20 | 212 (32.6) | 20 (9.0) | 158 (42.9) | 70 (23.7) | 460 (29.9) |
|  | Not applicable | 3 (0.46) | 90 (40.4) | 1 (0.3) | 14 (4.7) | 108 (7.0) |
| Number of hours worked per week | <35 | 149 (22.9) | 61 (27.4) | 72 (19.6) | 85 (28.8) | 367 (23.9) |
|  | 35-45 | 238 (36.6) | 121 (54.3) | 226 (61.4) | 158 (53.6) | 743 (48.3) |
|  | >45 hours | 245 (37.6) | 21 (9.4) | 62 (16.8) | 45 (15.3) | 373 (24.3) |
|  | Not applicable | 19 (2.9) | 20 (9.0) | 8 (2.2) | 7 (2.4) | 54 (3.5) |
| Redeployed during COVID-19 pandemic | No | 477 (73.3) | 160 (71.7) | 258 (70.1) | 224 (75.9) | 1119 (72.8) |
|  | Yes | 148 (22.7) | 47 (21.1) | 102 (27.7) | 64 (21.7) | 361 (23.5) |
|  | Not applicable | 26 (4.0) | 16 (7.2) | 8 (2.2) | 7 (2.4) | 57 (3.7) |
| If redeployed, received adequate training | No | 74 (11.4) | 23 (10.3) | 67 (18.2) | 27 (9.2) | 191 (12.4) |
|  | Yes | 77 (11.8) | 24 (10.8) | 34 (9.2) | 31 (10.5) | 166 (10.8) |
|  | Not applicable | 500 (76.8) | 176 (78.9) | 267 (72.6) | 237 (80.3) | 1180 (76.8) |
| Team understaffed during COVID-19 pandemic | Never | 224 (34.4) | 53 (23.8) | 81 (22.0) | 89 (30.2) | 447 (29.1) |
|  | 25% of the time | 163 (25.0) | 39 (17.5) | 92 (25.0) | 57 (19.3) | 351 (22.8) |
|  | 50% of the time | 88 (13.5) | 40 (17.9) | 63 (17.1) | 56 (19.0) | 247 (16.1) |
|  | 75% of the time | 33 (5.1) | 26 (11.7) | 40 (10.9) | 21 (7.1) | 120 (7.8) |
|  | All the time | 60 (9.2) | 26 (11.7) | 52 (14.1) | 38 (12.9) | 176 (11.5) |
|  | Not applicable | 83 (12.7) | 39 (17.5) | 40 (10.9) | 34 (11.5) | 196 (12.8) |
| Adequate PPE at workplace | No | 176 (27.0) | 45 (20.2) | 82 (22.3) | 66 (22.4) | 369 (24.0) |
|  | Yes | 443 (68.0) | 144 (64.6) | 269 (73.1) | 206 (69.8) | 1062 (69.1) |
|  | Not applicable | 32 (4.9) | 34 (15.2) | 17 (4.6) | 23 (7.8) | 106 (6.9) |
| Appropriate PPE use training | No | 213 (32.7) | 41 (18.4) | 104 (28.3) | 99 (33.6) | 457 (29.7) |
|  | Yes | 384 (59.0) | 84 (37.7) | 226 (61.4) | 145 (49.2) | 839 (54.6) |
|  | Not applicable | 54 (8.3) | 98 (43.9) | 38 (10.3) | 51 (17.3) | 241 (15.7) |
| Work-related practices a source of stress | No | 137 (21.0) | 42 (18.8) | 59 (16.0) | 58 (19.7) | 296 (19.3) |
|  | Yes | 495 (76.0) | 153 (68.6) | 301 (81.8) | 229 (77.6) | 1178 (76.6) |
|  | Not applicable | 19 (2.9) | 28 (12.6) | 8 (2.2) | 8 (2.7) | 63 (4.1) |

| Supplemental Table 3a. Frequency of COVID symptoms, results of COVID tests and number of days off work due to suspected COVID symptoms in all participants at baseline. | | | | | | |
| --- | --- | --- | --- | --- | --- | --- |
| Evidence of COVID-19 at baseline | **Response** | **Medical Doctor**  **N = 607 (%)** | **HCA or other**  **N = 208 (%)** | **Nurses or Midwives**  **N = 355 (%)** | **AHP or Pharmacist**  **N = 285 (%)** | **Overall**  **N = 1455 (%)** |
| Symptoms suggestive of COVID-19 infection in past few months | No | 409 (67.4) | 153 (73.6) | 230 (64.8) | 202 (70.9) | 994 (68.3) |
|  | Yes | 191 (31.5) | 53 (25.5) | 124 (34.9) | 83 (29.1) | 451 (31.0) |
|  | Prefer not to disclose | 7 (1.2) | 2 (1.0) | 1 (0.3) | 0 (0.0) | 10 (0.7) |
| Evidence of COVID-19 from either viral swab or antibody test | No | 504 (83.0) | 174 (83.7) | 276 (77.7) | 232 (81.4) | 1186 (81.5) |
|  | Yes | 103 (17.0) | 34 (16.3) | 79 (22.3) | 53 (18.6) | 269 (18.5) |
| Number of days off work due to symptoms suspected of COVID-19 | None | 418 (68.9) | 155 (74.5) | 236 (66.5) | 215 (75.4) | 1024 (70.4) |
|  | 1-7 days | 116 (19.1) | 28 (13.5) | 55 (15.5) | 41 (14.4) | 240 (16.5) |
|  | 8-14 days | 49 (8.1) | 11 (5.3) | 36 (10.1) | 19 (6.7) | 115 (7.9) |
|  | >14 days | 24 (4.0) | 14 (6.7) | 28 (7.9) | 10 (3.5) | 76 (5.2) |

| Supplemental Table 3b. Frequency of COVID symptoms, results of COVID tests and number of days off work due to suspected COVID symptoms at follow-up. | | | | | | |
| --- | --- | --- | --- | --- | --- | --- |
| Evidence of COVID-19 at follow-up | **Response** | **Medical Doctor**  **N = 261 (%)** | **HCA or other**  **N = 108 (%)** | **Nurses or Midwives**  **N = 197 (%)** | **AHP or Pharmacist**  **N = 170 (%)** | **Overall**  **N = 736** |
| Symptoms suggestive of COVID-19 infection in past few months* | No | 160 (62.5) | 71 (67.0) | 117 (59.7) | 113 (67.3) | 461 (63.5) |
|  | Yes | 96 (37.5) | 35 (32.4) | 78 (39.8) | 54 (32.1) | 263 (36.2) |
|  | Prefer not to disclose | 0 (0.0) | 0 (0.0) | 1 (0.5) | 1 (0.6) | 2 (0.3) |
| Evidence of COVID-19 from either viral swab or antibody test | No | 198 (75.9) | 88 (81.5) | 142 (72.1) | 129 (75.9) | 557 (75.7) |
|  | Yes | 63 (24.1) | 20 (18.5) | 55 (27.9) | 41 (24.1) | 179 (24.3) |
| *Note.* * denotes n = 726 due to incomplete data: Medical doctors = 256, HCA or other = 106, Nurses or Midwives = 196, and AHPs = 168. | | | | | | |

| **Supplemental Table 4.** Mean values for the change in mental health, wellbeing, and burnout scores over the study period, stratified by professional role. | | | | |
| --- | --- | --- | --- | --- |
|  | **Professional role** | **Mean** | **Standard Deviation** | **Range** |
| **PHQ-9** | Medical doctor (n = 247 | 0.55 | 4.42 | -18 to 16 |
|  | HCA or other (n = 100) | -0.34 | 4.25 | -11 to 14 |
|  | Nurse and midwives (n = 184) | 0.58 | 5.19 | -16 to 18 |
|  | AHPs (n = 164) | 0.01 | 3.76 | -13 to 10 |
| **GAD-7** | Medical doctor (n = 247) | -0.33 | 4.16 | -14 to 17 |
|  | HCA or other (n = 99) | -0.21 | 3.64 | -15 to 8 |
|  | Nurse and midwives (n = 184) | 0.74 | 4.67 | -18 to 21 |
|  | AHPs (n = 164) | 0.21 | 4.43 | -14 to 14 |
| **ISI** | Medical doctor (n = 246) | 0.30 | 3.48 | -11 to 10 |
|  | HCA or other (n = 99) | -0.08 | 4.82 | -16 to 12 |
|  | Nurse and midwives (n = 184) | 0.22 | 4.96 | -17 to 28 |
|  | AHPs (n = 163) | -0.21 | 4.79 | -16 to 14 |
| **SWEMWBS** | Medical doctor (n = 245) | 0.30 | 3.48 | -11 to 10 |
|  | HCA or other (n = 99) | -0.21 | 3.74 | -9 to 14 |
|  | Nurse and midwives (n = 181) | -0.85 | 4.09 | -19 to 12 |
|  | AHPs (n = 162) | -0.28 | 3.29 | -12 to 12 |
| **Combined burnout domains** | Medical doctor (n = 245) | 0.24 | 2.42 | -8 to 8 |
|  | HCA or other (n = 98) | 0.26 | 3.00 | -6 to 8 |
|  | Nurse and midwives (n = 179) | 1.20 | 2.75 | -7 to 10 |
|  | AHPs (n = 162) | 0.56 | 2.54 | -6 to 10 |
| *Note.* Change in scores for each measure are calculated by subtracting the raw score at follow-up from the baseline raw score.  * Adjusted for age, gender, time since COVID peak, highest level of education, relationship status, number living in household, current diagnosis of mental health condition, current diagnosis of physical health condition, and part-time/full-time working status. | | | | |

| **Supplemental Table 5**. Coefficients between worry items and mental health scores in all participants at baseline (n, 1386) and follow-up (n, 685). | | | | | | |
| --- | --- | --- | --- | --- | --- | --- |
| Variable | Worry about health | Worry about PPE | Worry about family | Worry about training | Worry about supervision | Worry about redeployment |
| *Baseline* |  |  |  |  |  |  |
| PHQ-9 score | 0.23* | 0.24* | 0.23* | 0.25* | 0.21* | 0.22* |
| GAD-7 score | 0.28* | 0.21* | 0.26* | 0.25* | 0.25* | 0.21* |
| ISI-7 score | 0.25* | 0.22* | 0.21* | 0.24* | 0.21* | 0.19* |
| SWEMWBS score | -0.22* | -0.20* | -0.18* | -0.26* | -0.25* | -0.24* |
| Emotional exhaustion score | 0.16* | 0.21* | 0.20* | 0.25* | 0.22* | 0.16* |
| Depersonalisation score | 0.07* | 0.14* | 0.13* | 0.19* | 0.19* | 0.11* |
| *Follow-up* |  |  |  |  |  |  |
| PHQ-9 score | 0.23* | 0.22* | 0.25* | 0.25* | 0.25* | 0.15* |
| GAD-7 score | 0.26* | 0.20* | 0.27* | 0.25* | 0.28* | 0.17* |
| ISI-7 score | 0.24* | 0.18* | 0.22* | 0.23* | 0.23* | 0.16* |
| SWEMWBS score | -0.20* | -0.15* | -0.19* | -0.20* | -0.25* | -0.17* |
| Emotional exhaustion score | 0.21* | 0.18* | 0.22* | 0.22* | 0.23* | 0.14* |
| Depersonalisation score | 0.12* | 0.16* | 0.14* | 0.18* | 0.17* | 0.08 |
| *Note. ** denotes significance to p <0.01, otherwise at p < 0.05. Listwise deletion used for missing data. | | | | | | |

| Supplemental Table 6a. Logistic regressions comparing risk of mental health outcomes in senior doctors (n, 437) compared to other doctors (regardless of training level; n, 214) at baseline. | | | |
| --- | --- | --- | --- |
| Outcome | **Role** | **Adjusted ORs (95% CI)** | **P-value** |
| Major depressive disorder | Doctors | Reference | - |
|  | Senior doctors | 0.96 (0.52 to 1.77) | 0.90 |
| Generalised anxiety disorder | Doctors | Reference | - |
|  | Senior doctors | 1.14 (0.59 to 2.19) | 0.69 |
| Clinical insomnia | Doctors | Reference | - |
|  | Senior doctors | 1.03 (0.49 to 2.14) | 0.95 |
| Possible or probable depression or anxiety (SWEMWBS) | Doctors | Reference | - |
|  | Senior doctors | 1.17 (0.65 to 2.10) | 0.61 |
| Burnout (Emotional Exhaustion) | Doctors | Reference | - |
|  | Senior doctors | 0.85 (0.53 to 1.38) | 0.52 |
| Burnout (Depersonalisation) | Doctors | Reference | - |
|  | Senior doctors | 1.05 (0.55 to 2.02) | 0.87 |
| *Note*. Adjusted for age and gender identity. Senior doctors included consultants, GPs, or staff specialists. Other doctors included clinical fellow/junior doctors, core trainees, foundation doctors, or higher specialist trainees. Total participants varied for each adjusted model: major depressive disorder = 575, generalised anxiety disorder = 597, clinical insomnia = 575, SWEMWBS = 584, emotional exhaustion and depersonalisation = 583. | | | |

| Supplemental Table 6b. Logistic regressions comparing risk of mental health outcomes in senior doctors (n, 179) compared to other doctors (regardless of training level; n, 82) at follow-up. | | | |
| --- | --- | --- | --- |
| Outcome | **Role** | **Adjusted ORs (95% CI)** | **P-value** |
| Major depressive disorder | Doctors | Reference | - |
|  | Senior doctors | 0.56 (0.24 to 1.34) | 0.19 |
| Generalised anxiety disorder | Doctors | Reference | - |
|  | Senior doctors | 0.55 (0.21 to 1.42) | 0.22 |
| Clinical insomnia | Doctors | Reference | - |
|  | Senior doctors | 0.93 (0.28 to 3.10) | 0.91 |
| Possible or probable depression or anxiety (SWEMWBS) | Doctors | Reference | - |
|  | Senior doctors | 0.78 (0.34 to 1.83) | 0.58 |
| Burnout (Emotional Exhaustion) | Doctors | Reference | - |
|  | Senior doctors | 1.29 (0.60 to 2.79) | 0.51 |
| Burnout (Depersonalisation) | Doctors | Reference | - |
|  | Senior doctors | 1.16 (0.46 to 2.91) | 0.75 |
| *Note*. Adjusted for age and gender identity. Senior doctors included consultants, GPs, or staff specialists. Other doctors included clinical fellow/junior doctors, core trainees, foundation doctors, or higher specialist trainees. Total participants varied for each adjusted model: major depressive disorder = 254, generalised anxiety disorder = 240, clinical insomnia = 239, SWEMWBS = 252, emotional exhaustion = 252, and depersonalisation = 250. | | | |
